# Supplementary material for: The hippocampus dissociates present from past and future goals
Source: Nat Commun. 2024 Jun 6;15:4815. doi: 10.1038/s41467-024-48648-9 (PMC11156658; doi:10.1038/s41467-024-48648-9)
Supplement: Supplementary file 1 — Supplementary Information [file 41467_2024_48648_MOESM1_ESM.pdf]

The hippocampus dissociates present from past and future goals

SUPPLEMENTARY INFORMATION

*Alison Montagrin[1,2,3], *Denise E. Croote[1], Maria Giulia Preti[4,5,6], Liron Lerman[7], Mark G. Baxter[1], Daniela Schiller[1,8]

[1]The Nash Family Department of Neuroscience, Icahn School of Medicine at Mount Sinai, New York, NY, 10029, USA
[2]Department of Neuroscience, University of Geneva, Geneva, 1202, Switzerland
[3]Swiss Center for Affective Sciences (CISA), University of Geneva, 1202 Geneva, Switzerland
[4]CIBM Center for Biomedical Imaging, Switzerland
[5]Neuro-X Institute, École Polytechnique Fédérale de Lausanne (EPFL), Lausanne, Switzerland
[6]Department of Radiology and Medical Informatics, University of Geneva (UNIGE), Geneva, Switzerland
[7]Sector 5 Digital, New York, NY, 10018, USA
[8]Department of Psychiatry, Icahn School of Medicine at Mount Sinai, New York, NY, 10029, USA

*these authors contributed equally

Corresponding Authors:

Alison Montagrin
University of Geneva, Geneva, 1202, Geneva
Email: alison.montagrin@unige.ch
Tel: +41 22 379 07 51

Daniela Schiller
Icahn School of Medicine at Mount Sinai, New York, NY 10029
Email: daniela.schiller@mssm.edu
Tel: 212-824-8977

Supplementary Method

R Software

In our analyses, data manipulation and transformation were conducted using the dplyr[1], plyr[2], and tidyr[3] packages. Visualizations were created with ggplot2[4], and complex data arrangements were facilitated by gridExtra[5]. For statistical modeling, we utilized lmerTest[6] and car[7], with post hoc analyses conducted via emmeans[8]. The effectsize package[9] package was used to calculate effect sizes.

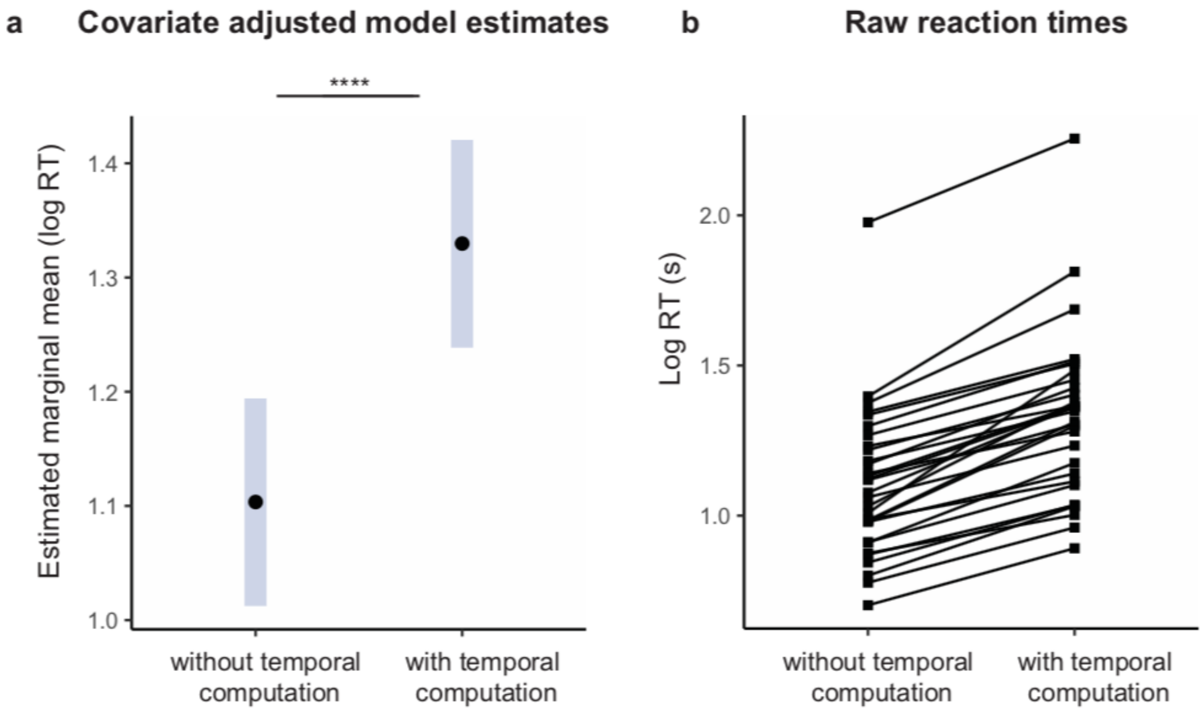

<u>Supplementary Figure 1</u>. Participants took longer to process goals that involved a temporal computation. We explored the relationship between participants' log transformed reaction times and trial type using a linear mixed model. We compared trials with a temporal computation (distant future, near future, near past, distant past) to those without a temporal computation (current, always, never). Log transformed[1] reaction time was significantly associated with trial type (Type II ANOVA; $F(1,\ 3422.7) = 455.464$, $p<0.001$). **(a)** A *post hoc* examination of the difference between the two estimated marginal means revealed that temporally removed goals took significantly longer to process (0.226 ± 0.011, $Z = 21.121$, $p<0.001$ in a two-tailed test). Error bars = 95% CI. **(b)** This effect was present at the individual subject level when examining participants' average log reaction times. ****$p<0.001$.

[1] This transformation aimed to address skewness and stabilize variance across conditions, making the data more suitable for parametric analysis.

a

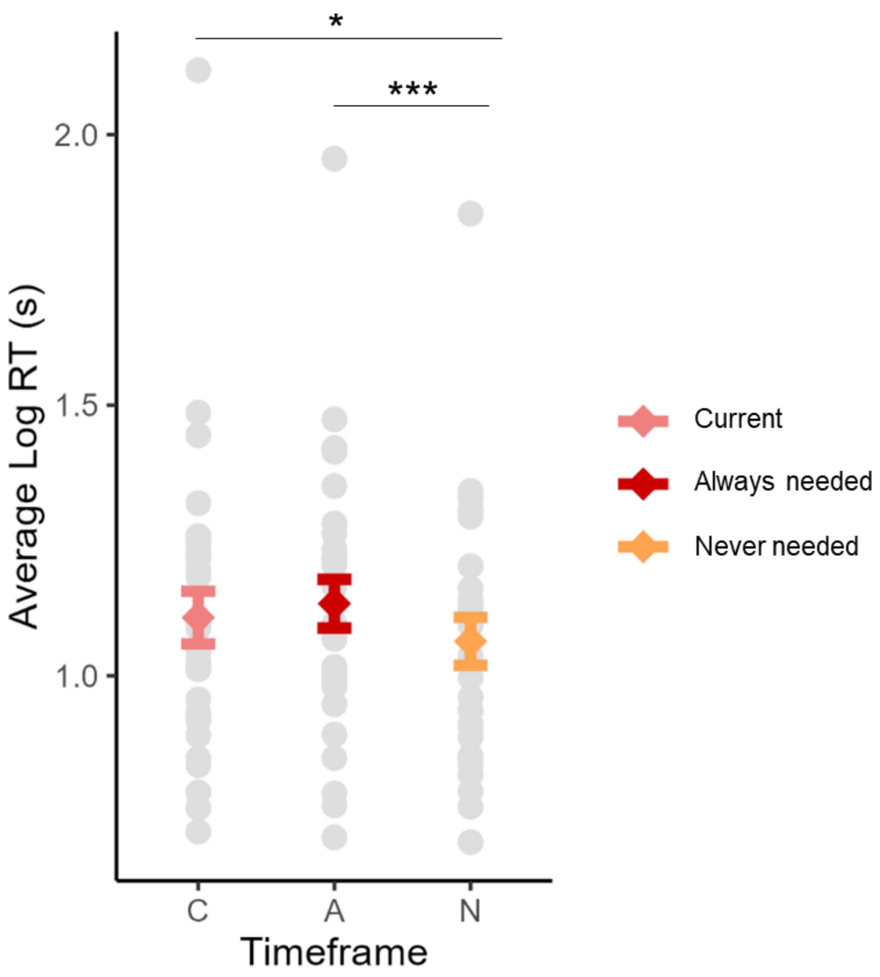

<u>Supplementary Figure 2</u>. Post hoc reaction time comparisons for current versus always needed (AN) and never needed (NN) Post hoc comparisons of the estimated marginal means revealed that current (0.044±0.018, t=2.471, p=0.036 in a two-tailed test) and AN (-0.073±0.018, t=-4.055, p<0.001 in a two-tailed test) goals were processed slower than NN goals. Error bars = 95% confidence Interval (CI). C, current goals; A, always needed goals; N, never needed goals. The p values were corrected for multiple comparisons using the Tukey method for comparing a family of 3 estimates. Degrees of freedom were approximated using the Satterthwaite method. *** p=0.001, *p<0.05.

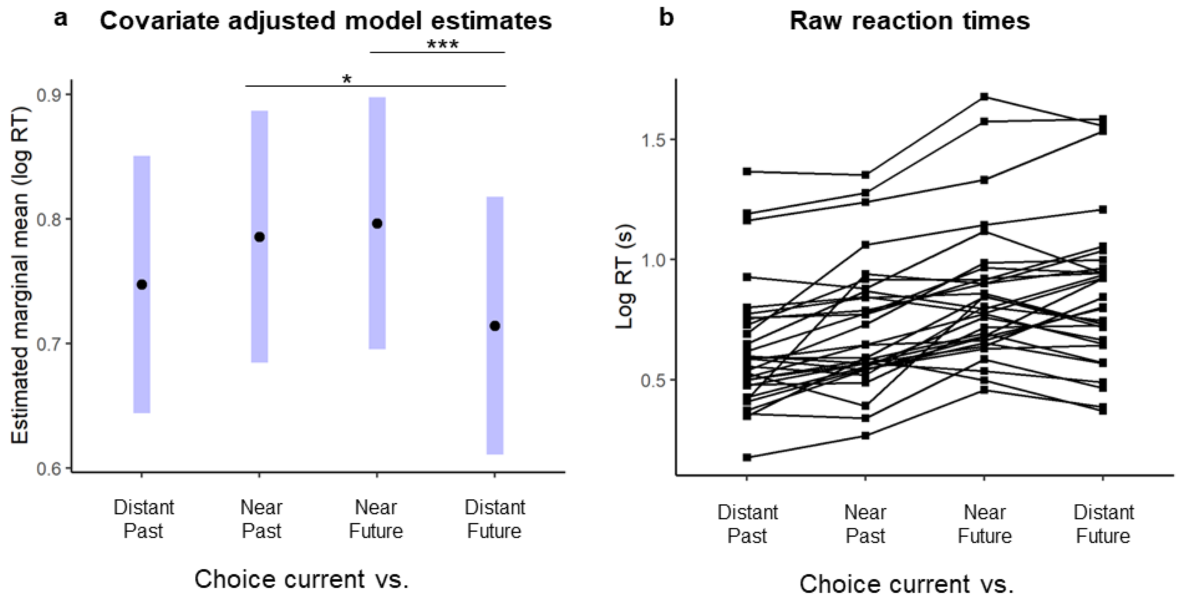

Supplementary Figure 3. Additional choice trials: Participants took longer to choose between current and near future or near past goals than current and distant future goals. (a) *Post hoc* comparisons of the estimated marginal means revealed that when participants had to choose between current and distant future goals, they processed this choice faster than when they had to choose between current and near future goals and current and near past goals. Error bars = 95% confidence Interval (CI). (b) Individual participants' average log reaction time for each of the temporal conditions against current. *** p=0.001, *p<0.05.

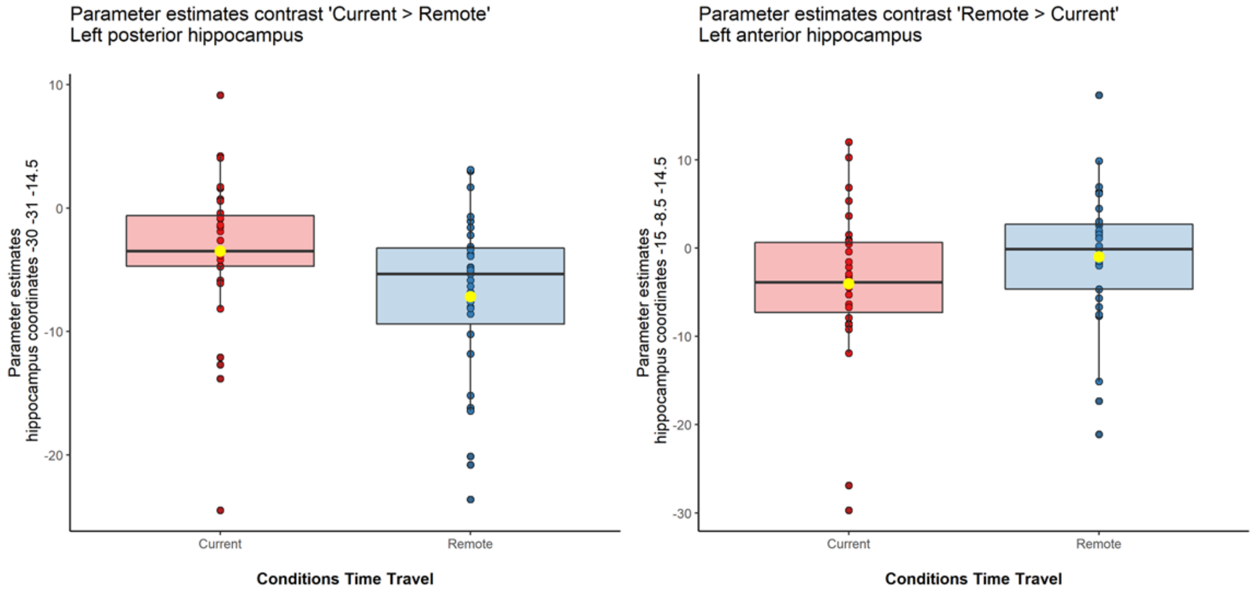

<u>Supplementary Figure 4</u>. Boxplot of parameter estimates of left hippocampus separately for two coordinates. On the left panel, posterior hippocampus coordinates extracted from first level for the condition “Current” and “Remote” from the peak voxel [-30 -31 -14.5], (current condition: *Mean* = -3.51, *SEM* = 1.11; remote condition: *Mean* = -7.18, *SEM* = 1.22). On the right panel, anterior hippocampus coordinated extracted from first level for the condition Remote and Current, from the peak voxel [-15 -8.5 -14.5], (current condition: *Mean* = -4.05, *SEM* .1.54; remote condition: *Mean* = -1.01, *SEM* = 1.38). Data points represent individual beta values among participants. Yellow points indicate the mean. Boxplots show the interquartile range (IQR), with whiskers extending to the highest points within 1.5 times the IQR of the upper and lower quartiles, illustrating the distribution of parameter estimates.

a Whole brain activation for temporally removed and current goals

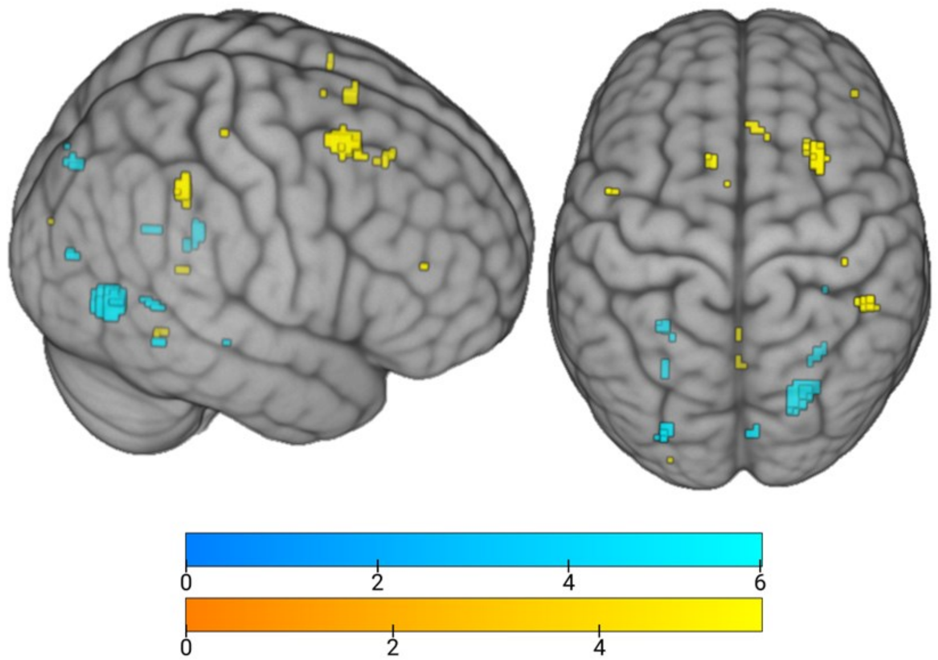

<u>Supplementary Figure 5</u>. Whole brain GLM results for temporally removed and current goals. Goals that were removed in time activated more anterior regions of the brain, while current goals activated more posterior regions of the brain. **(a)** A contrast comparing the Remote (distant future + near future + distant past + near past) > Current are overlaid in yellow. A contrast comparing the Current > Remote (distant future + near future + distant past + near past) are overlaid in blue. All z-statistic images were thresholded parametrically using maximum height thresholding (FWE voxel-wise correction, *p*=0.025). Contrasts maps were overlaid and rendered onto a 3-dimensional MNI 152 brain using MRIcroGL. Color bars reflect the thresholded z-statistic scores [z=0-6].

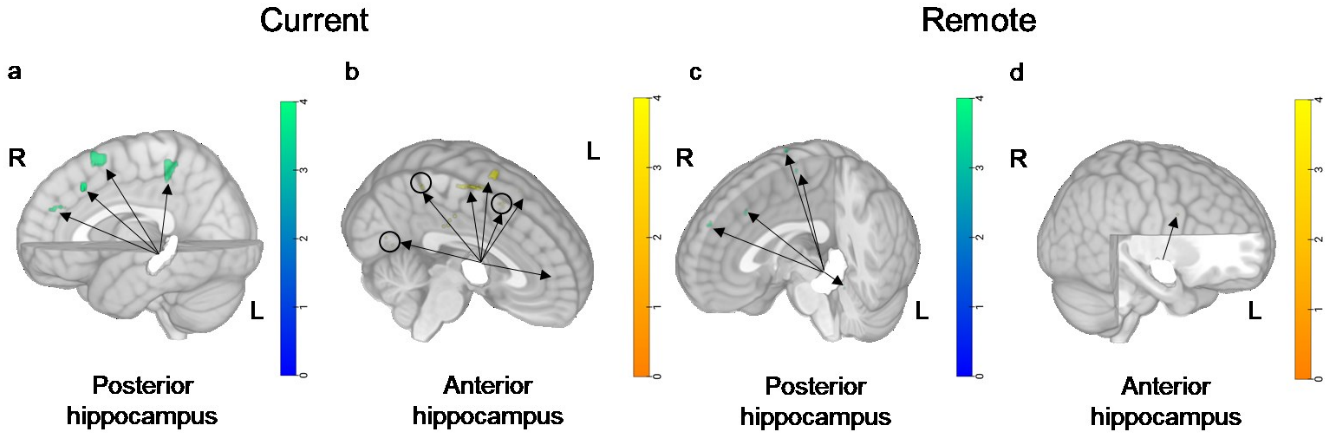

<u>Supplementary Figure 6.</u> Analysis of functional connectivity for temporally removed and current goals. We performed a General Psychophysiological Interaction (gPPI) analyses using the left anterior and posterior hippocampus as seed regions. For the goals that were current (a) the posterior part of the hippocampus activated the frontal and parietal lobes in the right hemisphere, whereas (b) the anterior part activated the frontal, parietal and occipital lobes in the left hemisphere. For the goals that were removed in time (c) the posterior part of the hippocampus activated the frontal and parietal lobes, cingulate gyrus, and cerebellum in the right hemisphere, whereas (d) the anterior part of the hippocampus activated the frontal lobe in the right hemisphere. All z-statistic images were thresholded parametrically using maximum height thresholding (FWE voxel-wise correction, p=0.025). Contrasts maps were overlaid and rendered onto a 3-dimensional MNI 152 brain using MRIcroGL. Color bars reflect the thresholded z-statistic scores [z=0-4]. R, right; L, left.

<u>Supplementary Note 1.</u> We found that when goals were in the current moment, the left posterior hippocampus showed stronger association with regions in the right hemisphere, while the anterior part was primarily linked to regions in the left hemisphere. However, when goals were removed in time, both the left posterior and anterior hippocampus exhibit a greater association with regions in the right hemisphere. This pattern suggests a dynamic shift in hemispheric involvement based on the temporal context of the goals. These results align with the notion that different brain regions coordinate activity in response to specific cognitive demands and suggest that dynamic brain networks may be recruited during various types of memory processing (i.e. current versus remote goals). Further investigation is needed to understand the nuanced nature in which subregions of the hippocampus interact with bilateral cortical regions when processing temporal distance.

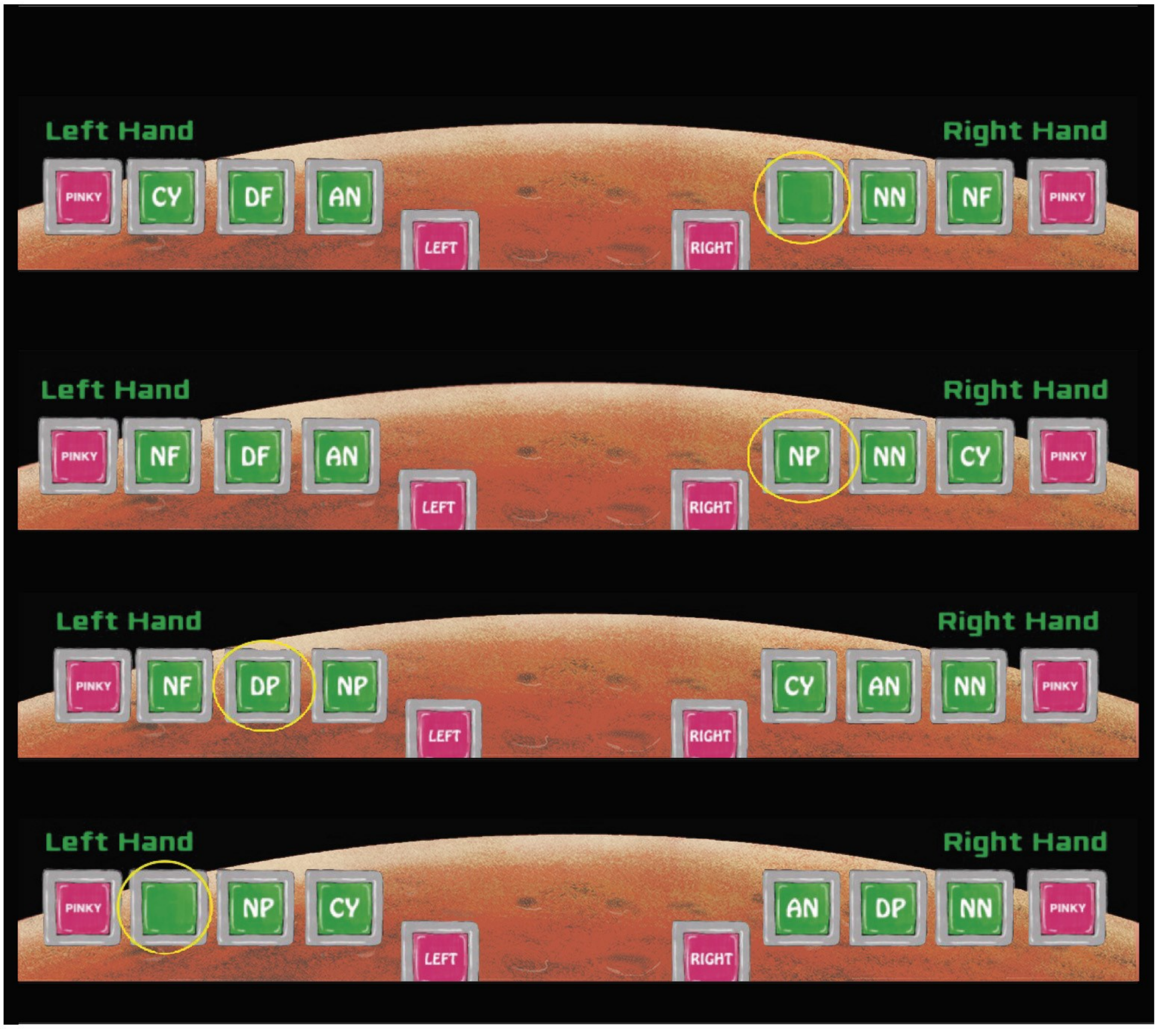

Supplementary Figure 7. Game buttons. Participants selected their responses using hand pads attached to their right and left hands. The button options remained the same within a year and rotated on the screen after every trial to prevent the reaction times for a condition from being influenced by the ease of using one finger over another. Across years, the buttons present on the screen changed depending on whether past or future options were applicable.

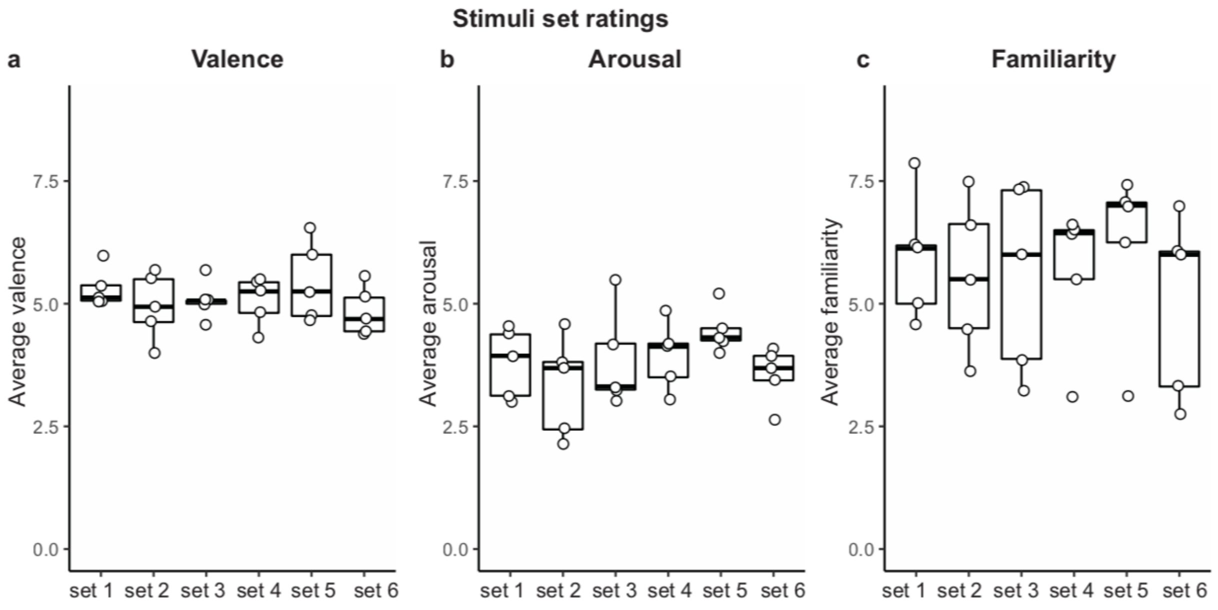

Supplementary Figure 8. Stimuli selection. Goals were selected using an online survey in an independent sample. The goals selected belonged to 5 categories (space shuttle, space suit, food, exercise, and recreational activity) and one goal from each category was randomly assigned to a set. There were no differences in **(a)** valence (Kruskal-Wallis rank sum test; $X^2_{(5)}$ =3.02, *p*=0.70) **(b)** arousal (Kruskal-Wallis rank sum test; $X^2_{(5)}$ =6.11, *p*=0.30) or **(c)** familiarity (Kruskal-Wallis rank sum test; $X^2_{(5)}$ =2.05, *p*=0.84) across each set of goals. Each goal set stayed as a unit. However, the timeframe that the goal set was assigned to (year 1, year 2, year, 3, year 4, always, never) varied by task version. Box plots; center line=median, box limits=Q1 and Q3, whiskers= smallest/largest value no further than 1.5x IQR. Individual data points= average rating for each goal stimulus in the set.

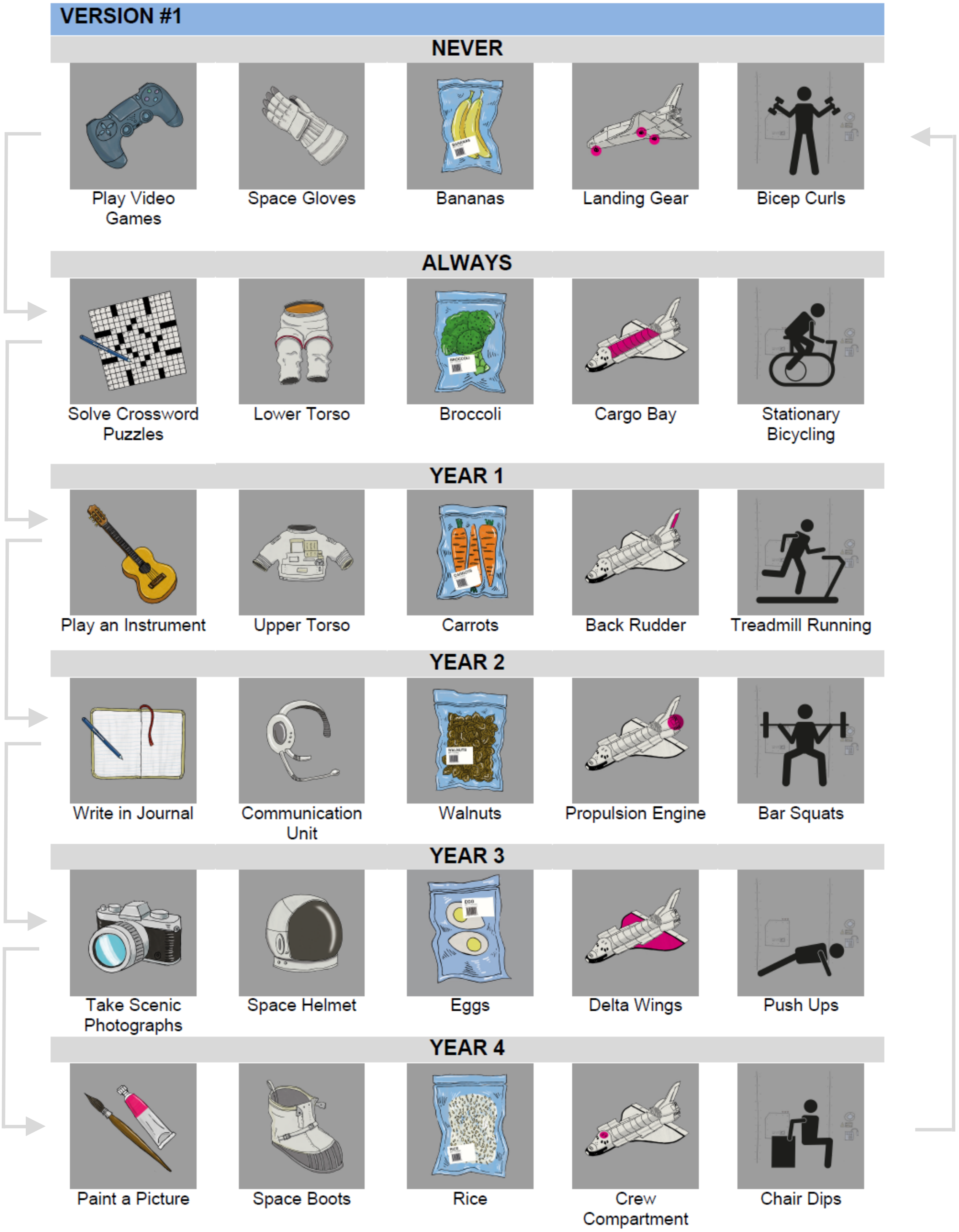

<u>Supplementary Figure 9</u>. Stimuli and version visualization. Stimuli and timeframe assignments for version 1 of the task. The arrows indicate how the stimuli transitioned across the 6 versions of the task. Each set of 5 goals stayed as a unit and was assigned to every timeframe (year 1, year 2, year 3, year 4, always, never) across the different versions.

Region of interest	Image	Application
Left hippocampus	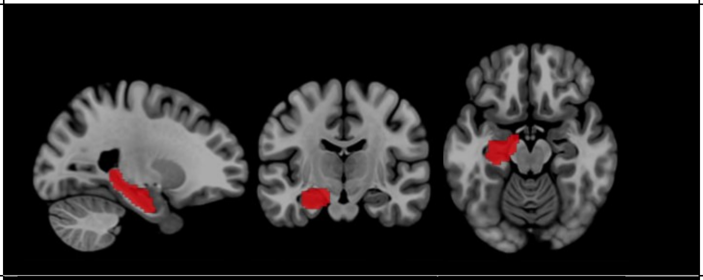	Applied at the group level during the region of interest analysis.
Right hippocampus	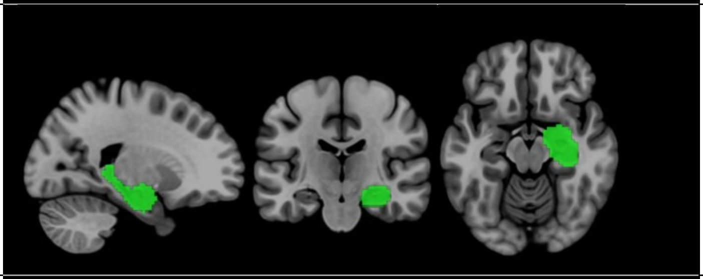	Applied at the group level during the region of interest analysis.
Whole brain gray matter mask	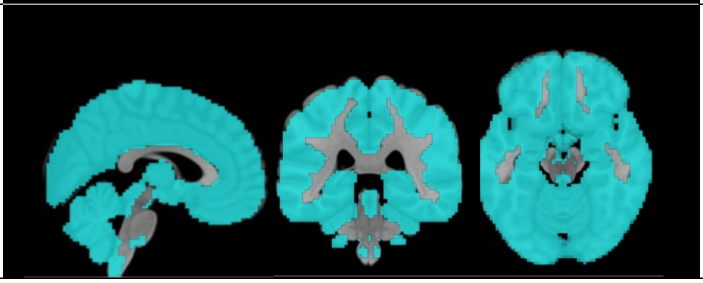	Applied at the group level during the whole brain analysis.

<u>Supplementary Figure 10</u>. Functional MRI masks. All masks are in the $2.5mm^3$ MNI space and overlaid a standard brain image, MNI 152 T1 $2mm^3$.

Contrast	Voxel size	Hemisphere	Hippocampus Axis	Z-stat	Coordinates in mm (MNI)		
AN > Remote	13	L	Posterior	3.95	-35	-23.5	-12
	1	L	Anterior	3.75	-13.5	-14.5	-32.5
Remote > AN	-	-	-	-	-	-	-
AN > Current	19	L	Anterior	4.03	-12.5	-8.5	-14.5
	1	L	Posterior	3.57	-32.5	-28.5	-14.5
Current > AN	-	-	-	-	-	-	-
AN > NN	-	-	-	-	-	-	-
NN > AN	-	-	-	-	-	-	-
NN > Remote	-	-	-	-	-	-	-
Remote > NN	175	L	Posterior	4.81	-27.5	-23.5	-14.5
NN > Current	125	L	Anterior	5.01	-17.5	-11	-12
Current > NN	-	-	-	-	-	-	-

Supplementary Table 1. Coordinates of peak activation for remote, current, always needed and never needed goals in the hippocampus. Always needed (AN) goals activated both the posterior and anterior regions. This suggests that, unlike Current trials, Always trials are not only relevant to the current moment, but also to the past and to the future, and thus may have a broader representation. The never needed (NN) goals exhibited a resemblance to the remote goals, revealing activity in the anterior part of the hippocampus. The table reports the number of significant voxels in the cluster, maximum z-statistic within the cluster, and the x, y, z location of the maximum intensity voxel for each contrast. Coordinates are reported in Montreal Neurological Institute (MNI) space. All statistical maps were corrected for multiple comparisons using maximum height thresholding (FWE voxel-wise correction, p=0.025). AN, always needed goals; NN, never needed goals.

	Mars year 1	Mars year 2	Mars year 3	Mars year 4
Current	5	5	5	5
Always	5	5	5	5
Never	5	5	5	5
Distant future	10	5		
Near future	5	5	5	
Near past		5	5	5
Distant past			5	10

Supplementary Table 2. Distribution of game trials. Participants were presented with 120 trials during the game. The numbers in the boxes reflect the number of trials for the given temporal condition during that game year. There were 20 trials each for the current, always, and never conditions, and 15 trials each for the temporally removed conditions. Current, always, and never trials were presented across all four game years. Distant future trials were presented in the first two game years, near future trials presented in the first three, near past trials presented in the last three, and distant past trials presented in the last two game years.

a

Contrast	Estimate	SE	df	t.ratio	P value	Significance
current vs. distant future	-0.227	0.023	2294	-10.05	<0.0001	***
current vs. near future	-0.24	0.021	2294	-11.62	<0.0001	***
current vs. near past	-0.28	0.021	2294	-13.54	<0.0001	***
current vs. distant past	-0.141	0.022	2294	-6.39	<0.0001	***
distant future vs. distant past	-0.086	0.029	2294	-2.95	0.026	**
near future vs. near past	0.039	0.023	2294	1.67	0.451	
near future vs. distant past	-0.098	0.025	2294	-3.81	0.001	**
near past vs distant past	-0.137	0.022	2294	-6.03	<0.0001	***

b

**Timeframe**	**emmean**	**SE**	**df**	**lower.CL**	**upper.CL**
current	1.11	0.050	33.2	1.01	1.21
distant past	1.25	0.051	37.3	1.15	1.35
near past	1.39	0.051	35.5	1.29	1.49
near future	1.35	0.051	35.5	1.25	1.45
distant future	1.33	0.051	37.8	1.23	1.44

Supplementary Table 3. *Post hoc* reaction time comparisons for temporal conditions goals. **(a)** We modeled participants log transformed reaction times against temporal condition, with levels for distant future, near future, current, near past, and distant past trials. Participants processed current goals more quickly than all temporally removed past and future goals when compared individually via *post hoc* contrasts. Participants processed distant past goals more quickly than all temporally removed past and future goals when compared individually via *post hoc* contrasts. **(b)** The estimates presented in the table result from *post hoc* comparisons of the estimated marginal means for each condition. Each estimate included by its standard error (SE), degrees of freedom (df), and lower (lower.CL) and upper (upper.CL) bounds for the 95% confidence interval. The *p* values were corrected for multiple comparisons using the Tukey method for comparing a family of 5 estimates. Degrees of freedom were approximated using the Satterthwaite method.

ID	Regressors	Explanation
1-4	remote, current, always, never	Regressors denoting the timing for all correct trials in each temporal condition. Temporally removed conditions contained a maximum of 15 trials each, while current, always, and never conditions contained a maximum of 20 trials each.
5-8	year 1, year 2, year 3, year 4	Regressors denoting the timing for all trials in each game year, including: the task, additional choice, incorrect response, and reaction time outlier trials.
9-14	mp1, mp2, mp3, mp4, mp5, mp6	Six motion parameters (mp) capturing the translations and rotations.

Supplementary Table 4. GLM regressors for the general temporal analysis. Regressors included in the whole brain and hippocampal GLMs. These GLMs examined differences in neural activity between the temporally removed goals and the current goals. All trials were modeled with boxcars, where the duration was set to the participant's reaction time, and convolved with FEAT's gamma hemodynamic response function. Temporal derivatives and temporal filtering was applied to regressors 1-8. Motion parameter files represented the translation and rotation values outputted from the ME-ICA preprocessing pipeline.

ID	Regressors	Explanation
1-7	distant future, near future, near past, distant past, current, always, never	Regressors denoting the timing for all correct trials in each temporal condition. Temporally removed conditions contained a maximum of 15 trials each, while current, always, and never conditions contained a maximum of 20 trials each.
8-11	year 1, year 2, year 3, year 4	Regressors denoting the timing for all trials in each game year, including: the task, additional choice, incorrect response, and reaction time outlier trials.
12-17	mp1, mp2, mp3, mp4, mp5, mp6	Six motion parameters (mp) capturing the translations and rotations.

Supplementary Table 5. GLM regressors for the temporal gradient analysis. Regressors included in the whole brain and hippocampal GLMs. These GLMs examined differences in neural activity between the temporally removed goals and the current goals. All trials were modeled with boxcars, where the duration was set to the participant's reaction time, and convolved with FEAT's gamma hemodynamic response function. Temporal derivatives and temporal filtering were applied to regressors 1-11. Motion parameter files represented the translation and rotation values outputted from the ME-ICA preprocessing pipeline.

Contrast	Voxel size	Hemisphere	Hippocampus Axis	Z-stat	Coordinates in mm (MNI)		
Current > Distant Future	-	-	-	-	-	-	-
Current > Near Future	3	L	Posterior	4.13	-32.5	-26	-19.5
Current > Near Past	11	L	Posterior	4.04	-30	-31	-14.5
Current > Distant Past	1	L	Posterior	3.56	-32.5	-26	-19.5
Distant Future > Current	2	L	Anterior	3.73	-12.5	-13.5	-22
Near Future > Current	1	L	Anterior	3.55	-17.5	-11	-14.5
Near Past > Current	14	L	Anterior	4.6	-15	-8.5	-14.5
Distant Past > Current	10	L	Anterior	4.28	-12.5	-8.5	-14.5

Supplementary Table 6. Coordinates of peak activation for each temporally removed and current goals in the left hippocampus. Each temporally removed goals (Distant Future, Near Future, Near Past and Distant Past) activated the left anterior hippocampus, while current goals activated the left posterior hippocampus (except Current > Distant Future that show no activation). This table reports the number of significant voxels in the cluster, maximum z-statistic within the cluster, and the x, y, z location of the maximum intensity voxel for each contrast. Coordinates are reported in Montreal Neurological Institute (MNI) space. All statistical maps were corrected for multiple comparisons using maximum height thresholding (FWE voxel-wise correction, p=0.025).

Contrast	voxel size	z-max	Coordinates in mm			Hemisphere	Lobe	Region
**current > remote**	56	6.02	22.5	-68.5	-9.5	R	Occipital	Lingual Gyrus
	13	5.01	-30	-46	-7	L	Limbic/Occipital	Fusiform/Lingual Gyrus
	7	5.13	-27.5	-88.5	15.5	L	Occipital	Occipital Gyrus
	6	5.25	30	-53.5	-7	R	Occipital	Lingual Gyrus
	3	4.95	-27.5	-61	-9.5	L	Occipital	Lingual Gyrus
	3	5.02	7.5	-86	-2	R	Occipital	Calcarine
	2	4.93	30	-51	-22	R	Cerebellum	Culmen
	1	4.84	32.5	-28.5	-19.5	R	Limbic	Parahippocampal Gyrus
**remote > current**	33	5.52	30	11.5	58	R	Frontal	Middle Frontal Gyrus
	18	5.56	45	-41	48	R	Parietal	Inferior Parietal Lobule
	7	5.17	7.5	24	40.5	R	Frontal	Cingulate Gyrus
	7	5.32	-10	11.5	58	L	Frontal	Cingulate Gyrus
	3	4.93	0	-53.5	-34.5	L/R	Cerebellum	Vermis
	3	5.57	-50	-1	50.5	L	Frontal	Precentral Gyrus
	2	5.08	0	-48.5	-9.5	L/R	Cerebellum	Vermis
	1	4.83	-25	-96	-7	L	Occipital	Cuneus
	1	4.9	42.5	41.5	20.5	R	Frontal	Middle Frontal Gyrus
	1	4.84	-5	1.5	60.5	L	Frontal	Supplemental Motor Area
	1	4.83	40	-28.5	68	R	Frontal	Precentral Gyrus

<u>**Supplementary Table 7**</u>**. Whole brain activation coordinates for temporally removed goals.** Temporally removed (distant future, near future, distant past and near past) goals activated bilaterally more anterior regions of the brain, while current goals activated bilaterally more posterior regions of the brain. This table reports the number of significant voxels in the cluster, maximum z-statistic within the cluster, and the x, y, z location of the maximum intensity voxel for each contrast. Coordinates are reported in Montreal Neurological Institute (MNI) space. All statistical maps were corrected for multiple comparisons using maximum height thresholding (FWE voxel-wise correction, p=0.025).

a

Voxel size	Hemisphere	Region	Z-stat	Coordinates in mm
40	R	Frontal lobe	5.9	30 14 60.5
38	R	Parietal lobe	6.64	50 -43.5 50.5
12	L/R	Frontal lobe	5.41	0 26.5 43
9	R	Frontal lobe	5.39	43 42 22

b

Voxel size	Hemisphere	Region	Z-stat	Coordinates in mm
3	R	Frontal lobe	5,32	37.5 39 23
3	R	Cingulate gyrus	5	7.5 24 40.5
3	R	Parietal lobe	5,18	42.5 -31 65.5
2	R	Parietal lobe	5,19	45 -41 48
1	R	Cerebellum	5,01	0 -56 -34.5
1	R	Parietal lobe	5,05	-43.5 50.5 25.6

Supplementary Table 8. General Psychophysiological Interaction (gPPI) Analysis Results and Coordinates for Posterior Hippocampus Connectivity. This table reports the voxel coordinates, the maximum z-statistic within the cluster, and the x, y, z location of the significant functional connectivity observed in the posterior mask of the hippocampus for the **(a)** current condition, and **(b)** remote condition. Coordinates are reported in Montreal Neurological Institute (MNI) space. All statistical maps were corrected for multiple comparisons using maximum height thresholding (FWE voxel-wise correction, *p*=0.025).

a

Voxel size	Hemisphere	Region	Z-stat	Coordinates in mm
10	L	Frontal Lobe	5.39	-27.5 -1 63
7	L	Frontal lobe	5.45	-20 -16 53
6	L	Frontal lobe/Parietal lobe	6.11	-37,5 -26 38
6	L	Frontal lobe	5.3	-42.5 -6 25.5
4	R	Frontal lobe	5.4	22.5 -16 50.5
5	L	Parietal lobe	5,33	-20 -53.5 40.5
3	L	Frontal lobe	5,31	-25 -11 50.5
1	L	Occipital Lobe	4,99	-10 -71 -7
1	L	Occipital Lobe	4,91	-10 -76 -2
1	L	Frontal lobe	5,14	-55 4 30.5

b

Voxel size	Hemisphere	Region	Z-stat	Coordinates in mm
1	R	Frontal lobe	4.88	52.5 9 30.5

Supplementary Table 9. General Psychophysiological Interaction (gPPI) Analysis Results and Coordinates for Anterior Hippocampus Connectivity. This table reports the voxel coordinates, the maximum z-statistic within the cluster, and the x, y, z location of the significant functional connectivity observed in the anterior mask of the hippocampus for the **(a)** current condition, and **(b)** remote condition. Coordinates are reported in Montreal Neurological Institute (MNI) space. All statistical maps were corrected for multiple comparisons using maximum height thresholding (FWE voxel-wise correction, p=0.025).

<u>Supplementary Table 10</u>. Instructions.

**Day 1 – General introduction**<br>*Read to the participant*
In this experiment, you will be going on an expedition to Mars. This mission serves as the first manned mission to Mars and you will be traveling alone as the sole astronaut aboard the space shuttle! You will be on Mars for 4 years and during your stay you will have to abide by a very strict schedule. Each year you will have to complete a series of tasks. These tasks have been designed and planned by NASA's top engineers. They are in place to make sure that you maintain the space equipment we have on Mars and to make sure that you maintain your personal well-being. Each year you will be assigned different tasks and it is very important that you know when you have to complete each task. This computer module here will teach you what tasks you must complete and when, meaning in which year, you must complete them. If you are unable to learn all of the material, you will not be able to go on the mission to Mars. Throughout the module, you will be quizzed on your knowledge. You will start by learning the tasks you need to complete for the 1st year, then you will move onto the 2nd year and so on. For the second portion of the module, you will learn the same material, but in the reverse order. This module is very difficult, so concentrate as best you can and take breaks whenever you need to! Good luck!
**Day 1 – Training exercise**<br>*Presented on screen*
Hello astronaut in training. Welcome to NASA's training program. As you know, you will be spending 4 years on Mars. In order to survive, you must complete a series of tasks throughout the year. These tasks are in place to ensure that you, the astronaut, stay healthy and to maintain the space equipment we have on Mars. The tasks you need to complete fall under the following categories: space shuttle maintenance, spacesuit maintenance, personal nutrition, exercise, and creative time (we won't make you work all day!). Each year (year 1, year 2, year 3, year 4) you will need to maintain a part of the space shuttle, maintain a part of your spacesuit, eat a certain food, complete a certain exercise, and enjoy a certain creative activity. There will be some tasks that you never need to complete, and other tasks that you always need to complete. This module will teach you which tasks to complete during year 1, year 2, year 3, year 4, which you will always need to complete, and which you will never need to complete. If you have any questions please ask the experimenter now. If not, click to continue.
**Day 2 – Mock scanning session**<br>*Read to the participant before entering the mock scanner*
Welcome back and congratulations for passing phase 1. Today you will go on your expedition to Mars. This part of the experiment will take place inside the scanner. Today's session is expected to last approximately 2 hours. We will first complete a training exercise in the mock scanner, to get you acquainted with the scanning environment. The setup of the mock scanner is exactly the same as the real scanner, however, there is no actual magnet, so this is just to get you comfortable with the size of the scanner and the television screen. Once you are familiar, we will move to the real scanner to let you go to Mars!<br><br>The experiment will take you through your 4 years on Mars. You will be presented with the same tasks that you saw during the training yesterday, except today you will be asked whether you must complete them in the year in which you currently reside. You will start in the present and you will go from Year 1 to Year 2, from Year 2 to Year 3 and so on, if you are able to survive. It's very important that you think about each task, when you need to complete it, and what year you are currently in. In order to advance through your mission, you must complete two different tasks:<br>1. When one task is presented on the screen you will have to decide which year this task belongs to.

2. You will be shown two tasks on the screen and you will be asked to choose the one that is the most important to complete for the current year.

During the training, the tasks presented are not the ones that you learned yesterday; their purpose is to acquaint you with the game format.

During your first year on Mars, tasks can take place in the current year or in the future. Near future tasks must be completed in the next year, while distant future tasks must be completed in 2 plus years. Since you are in the first year, there is no past. You will see a task appear in the gray box and you will need to think about the year that you are in and when in time you need to complete this task. When you are in Year 1, you will see the following buttons appear on the screen. CY stands for current year, AN for always need, NN for never need, NF for near future and DF for distant future.

If you succeed and advance to Year 2, there are tasks that need to be completed in the current year, in the near future, and in the distant future. However, since time has passed there are also tasks that should have been completed in the near past, meaning 1 year in the past. You will now see different buttons on the screen. You will still see buttons for current year, always need, never need, near future, and distant future, however, now you will also see a button “NP” for near past.

If you advance to Year 3, tasks will need to be completed in the future and also should have been completed in the past. There will be an option for near future, “NF”, however, there will no longer be a distant future “DF” option. This is because you are only spending 4 years on Mars, therefore, there are no tasks that need to be completed 2 plus years in the future. For the past, there will now be two options, one for near past, meaning 1 year in the past, and another for distant past, denoted “DP”, for tasks that needed to be completed 2 plus years in the past.

Finally, if you advance to Year 4 there are no tasks that need to be completed in the future because this is your last year on Mars. There will only be tasks that need to be completed in the current year, or needed to be completed in the near or distant past. Therefore, you will see that the near future and distant future buttons no longer appear on the screen.

The buttons will always rotate locations on the screen, so it is important that you look at the task, think about which year you are currently in, decide when you need to complete the task, and then pay attention to which button you are pressing. Occasionally, you will be shown two images on the screen. Here we want you to think about which task will most benefit your current survival right now. If you want the option on the left, click the left thumb button. If you want the option on the right, click the right thumb button.

Day 2 – Task instructions

Read to the participant while in the mock scanner

Welcome to Mars! As you know, you will be spending 4 years here. To ensure your survival, you must complete a series of tasks each year. If you cannot complete these tasks, you will not accomplish your mission and you will not return to earth. Meaning, you must achieve the different tasks of the current year to move onto the next year.

These tasks are in place to ensure that you the astronaut:

1.) Stay healthy. A nutritionist has created a diet adapted to the environment on Mars. Adhering to this diet will ensure that your immune system will be able to protect you from foreign and harmful substances over the 4 years. Thus, your yearly diet has been developed to (1) ensure that your body will gradually adapt to the environment over the years and (2) ensure that you can perform the activities that are planned each year. Failing to comply with this diet will put your health at risk and decrease the chance that you will have the necessary strength to perform the tasks required.

2.) Do not lose muscles mass and bone density. The medical team has planned exercises to minimize reductions in bone density, muscle strength, and to maintain your overall physical performance. These exercises will help you build the muscles that you need to perform the shuttle maintenance tasks in the current year. If you are not correctly trained you won't have the muscles needed to perform the different tasks each year.
3.) Avoid stress. Recreational activities are an essential ingredient to your overall quality of life. As an astronaut you need to take a break from your very busy schedule. A cognitive psychologist has developed different free time activities for you to partake in over the 4 years. You cannot skip these free time activities. Otherwise you won't have sufficient psychological strength to perform and achieve your tasks.
4.) Maintain and fix the space shuttle. NASA engineers have been studying the shuttle for years and have predicted which elements of the shuttle will break and erode over the course of the 4 years on Mars. The engineers have carefully planned (depending on climatic conditions, sand storms etc.) when certain parts must be fixed. Failing to fix the different parts of the shuttle during the scheduled year will prevent your safe return to earth.
5.) Maintain and fix the spacesuit equipment. Specialists have spent years investigating and selecting the materials for your spacesuit that will best enable you to withstand Mars's hostile environment. They have carefully planned (depending on climatic conditions, battery duration etc.) when certain parts of your spacesuit must be exchanged. Failing to change or fix the appropriate parts of the spacesuit during the scheduled year will threaten your safety.

As you navigate through the years on Mars you will see an image of a task displayed on the screen. Press:

- "Current" if the task has to be completed during the current year.
- "Near Future" if the task has to be completed in the next year.
- "Distant Future" if the task has to be completed in two or three years.

As you progress through the years. Some tasks will no longer be relevant. Press:

- "Near Past" if the task was completed in the previous year.
- "Distant Past" if the task was completed two or three years ago.

Some of the tasks have to be completed every year, i.e. you will always complete them, while other tasks never need to be completed. Press:

- "Always" if the task is always completed.
- "Never" if the task is never completed.

Throughout each year, you will occasionally see two tasks presented on the screen and you will be asked to select one of the tasks. Choose the task that you feel will most benefit your survival at that time. Press:

- "Left" if you would like to select the task on the left.
- "Right" if you would like to select the task on the right.

Do you have any questions? Good luck!

	**Mars year 1 ------- Mars year 2 ------- Mars year 3 --------- Mars year 4**
Goals should be accomplished in **year 1**	Current ------------- Near past ----------- Distant past ---------- Distant past
Goals should be accomplished in **year 2**	Near future --------- Current ------------- Near past ------------- Distant past
Goals should be accomplished in **year 3**	Distant future ------ Near future ---------- Current --------------- Near past
Goals should be accomplished in **year 4**	Distant future ------ Distant future ------- Near future ---------- Current

Supplementary Table 11. Temporal trajectories. The same goals were presented each year, but their temporal distance changed as participants advanced through the game. The trajectories of the goals depended on the year in which they needed to be accomplished. For instance, goals that needed to be accomplished in year 3 started off as goals relevant in the distant future, transitioned to goals relevant in the near future when the participant moved to the 2nd year of their Mars mission, became presently relevant in the 3rd year, and transitioned to something participants accomplished in the near past in their 4th year. We chose this design, rather than employing naturalistic goals from participants' personal pasts and futures, to isolate temporal distance.

Version	Number of Participants
V1	6
V2	6
V3	5
V4	5
V5	4
V6	5

Supplementary Table 12. Game versions. Participants learned sets of 5 goals that needed to be accomplished in year 1, year 2, year, 3, year 4, always, and never. We created six versions of the task to counterbalance all sets of stimuli. In version 1 of the task, participants needed to accomplish a given set of goals in year 1, in version 2 of the tasks participants needed to accomplish those same goals in year 2, in version 3 they needed to be accomplished in year 3, and so on. This rotation method ensured that each set of goals was assigned to every category and that any differences observed during the task could not be attributed to a stimuli-year combination being more memorable than the others.

References

1 Wickam, H., François, R., Henry, L. & Müller, K. *dplyr: A Grammar of Data Manipulation*, <https://CRAN.R-project.org/package=dplyr> (2023).

2 Wickam, H. The Split-Apply-Combine Strategy for Data Analysis. *Journal of Statistical Software* **40**, 1-29 (2011).

3 Wickham, H., Vaughan, D. & Girlich, M. *tidyr: Tidy Messy Data.*, <https://CRAN.R-project.org/package=tidyr> (2023).

4 Wickham, H. *ggplot2: Elegant Graphics for Data Analysis*, 2016).

5 Auguie, B. *gridExtra: Miscellaneous Functions for "Grid" Graphics*, <https://CRAN.R-project.org/package=gridExtra> (2017).

6 Kuznetsova, A., Brockhoff, P. B. & Christensen, R. H. B. lmerTest Package: Tests in Linear Mixed Effects Models. *Journal of Statistical Software* **82** (2017). https://doi.org/10.18637/jss.v082.i13

7 Fox, J. & Weisberg, S. *An R Companion to Applied Regression*, <https://socialsciences.mcmaster.ca/jfox/Books/Companion/> (2019).

8 Russell V. Lenth (2023). emmeans: Estimated Marginal Means, aka Least-Squares Means. R package version 1.9.0. https://CRAN.R-project.org/package=emmeans

9 Ben-Shachar, M., Lüdecke, D. & Makowski, D. effectsize: Estimation of Effect Size Indices and Standardized Parameters. *Journal of Open Source Software* **5** (2020). https://doi.org/doi: 10.21105/joss.02815
