# Supplementary material for: The hippocampus dissociates present from past and future goals
Source: Nat Commun. 2024 Jun 6;15:4815. doi: 10.1038/s41467-024-48648-9 (PMC11156658; doi:10.1038/s41467-024-48648-9)
Supplement: Supplementary file 3 — Reporting Summary [file 41467_2024_48648_MOESM3_ESM.pdf]

nature portfolio

Corresponding author(s): Daniela Schiller, Alison Montagrin

Last updated by author(s): March 25, 2024

Reporting Summary

Nature Portfolio wishes to improve the reproducibility of the work that we publish. This form provides structure for consistency and transparency in reporting. For further information on Nature Portfolio policies, see our Editorial Policies and the Editorial Policy Checklist.

Statistics

For all statistical analyses, confirm that the following items are present in the figure legend, table legend, main text, or Methods section.

n/a	Confirmed	
☐	☒	The exact sample size (*n*) for each experimental group/condition, given as a discrete number and unit of measurement
☐	☒	A statement on whether measurements were taken from distinct samples or whether the same sample was measured repeatedly
☐	☒	The statistical test(s) used AND whether they are one- or two-sided<br>*Only common tests should be described solely by name; describe more complex techniques in the Methods section.*
☐	☒	A description of all covariates tested
☐	☒	A description of any assumptions or corrections, such as tests of normality and adjustment for multiple comparisons
☐	☒	A full description of the statistical parameters including central tendency (e.g. means) or other basic estimates (e.g. regression coefficient) AND variation (e.g. standard deviation) or associated estimates of uncertainty (e.g. confidence intervals)
☐	☒	For null hypothesis testing, the test statistic (e.g. *F*, *t*, *r*) with confidence intervals, effect sizes, degrees of freedom and *P* value noted<br>*Give P values as exact values whenever suitable.*
☒	☐	For Bayesian analysis, information on the choice of priors and Markov chain Monte Carlo settings
☐	☒	For hierarchical and complex designs, identification of the appropriate level for tests and full reporting of outcomes
☒	☐	Estimates of effect sizes (e.g. Cohen's *d*, Pearson's *r*), indicating how they were calculated

Our web collection on statistics for biologists contains articles on many of the points above.

Software and code

Policy information about availability of computer code

Data collection	The training task was programmed in E-prime 2.0 and the experimental task was programmed in Unity version 5.6.3f1.
Data analysis	Behavioral reaction time analyses were conducted in R v.3.6.0. Functional MRI data was preprocessed and analyzed using the ME-ICA pipeline (multi-echo independent components analysis, https://bitbucket.org/prantikk/me-ica) and the Functional MRI of the Brain Software Library (FSL vS.0.10). In our analyses, data manipulation and transformation were conducted using the dplyr, plyr, and tidyr packages. Visualizations were created with ggplot2, and complex data arrangements were facilitated by gridExtra. For statistical modeling, we utilized lmerTest and car, with post hoc analyses conducted via emmeans. The effectsize package package was used to calculate effect sizes.

For manuscripts utilizing custom algorithms or software that are central to the research but not yet described in published literature, software must be made available to editors and reviewers. We strongly encourage code deposition in a community repository (e.g. GitHub). See the Nature Portfolio guidelines for submitting code & software for further information.

Data

Policy information about availability of data

All manuscripts must include a data availability statement. This statement should provide the following information, where applicable:

- Accession codes, unique identifiers, or web links for publicly available datasets
- A description of any restrictions on data availability
- For clinical datasets or third party data, please ensure that the statement adheres to our policy

The dataset generated and analyzed during the current study is available in the Open Science Framework repository [https://doi.org/10.17605/OSF.IO/3KT98].

Research involving human participants, their data, or biological material

Policy information about studies with human participants or human data. See also policy information about sex, gender (identity/presentation), and sexual orientation and race, ethnicity and racism.

Reporting on sex and gender	Our study is not focused on gender or biological sex. We have collected the information for demographic purposes (16 males and 15 females).
Reporting on race, ethnicity, or other socially relevant groupings	Not collected. None of these were considered variables in the task.
Population characteristics	Healthy control participants. See additional details below.
Recruitment	Participants were recruited via flyers in the community and email advertisements at Mount Sinai. Interested participants contacted the study team and were sent a screening questionnaire outlining the age requirement (18-55) and language requirement (fluent in English). This document also outlined the study exclusions (neurological illness, psychiatric illness, and MRI incompatibilities).
Ethics oversight	The Institutional Review Board at the Icahn School of Medicine at Mount Sinai approved this experiment. Informed consent was obtained from all participants. Compliance with all relevant ethical regulations was ensured throughout the study and methods were carried out in accordance with relevant guidelines and regulations.

Note that full information on the approval of the study protocol must also be provided in the manuscript.

Field-specific reporting

Please select the one below that is the best fit for your research. If you are not sure, read the appropriate sections before making your selection.

☐ Life sciences ☒ Behavioural & social sciences ☐ Ecological, evolutionary & environmental sciences

For a reference copy of the document with all sections, see nature.com/documents/nr-reporting-summary-flat.pdf

Behavioural & social sciences study design

All studies must disclose on these points even when the disclosure is negative.

Study description	This study investigated how individuals keep track of the temporal distance to different personal goals. The experiment took place over two days. On the first day of the experiment participants learned a series of stimuli and on the second day of the experiment participants completed a task inside a functional MRI scanner. This study included quantitative behavioral data and neuroimaging data.
Research sample	Participants were recruited from the community surrounding the Icahn School of Medicine at Mount Sinai in New York, NY, USA. Thirty-four medically and psychiatrically healthy adults completed the two-day experiment and 31 (n=15 female) remained in the sample after MRI exclusions. This representative sample was chosen such that participants would represent the neurotypical brain and be able to undergo MRI scanning. The mean age was 27 years old, the standard deviation 4.5 years, and the range 19 to 36 years old.
Sampling strategy	Our recruitment goal was to have 36 participants complete the task, distributed across six task versions. 36 participants were randomly recruited and 34 completed the experimental protocol. Once enrolled in the study, participants were assigned to one of the six different versions of the task. Version assignments were pseudo-randomized such that a roughly equal number of males and females completed each version of the task. 31 subjects remained in the study after exclusions and a subject by version breakdown is available in the supplement. No statistical method was used to predetermine sample size. A sex analysis was not included given existing literature suggested that it would have no significant impact on the results of the study.
Data collection	The training paradigm was completed on a Windows Lenovo Think Pad (15.5in) on the first day of the experiment. On the second day of the experiment the neuroimaging data was acquired on a 7T Siemens Magnetom scanner (Erlangen, Germany) with a 32-channel

	head coil (Nova Medical, Wilmington, MA). The experimenters were aware of the hypotheses and experimental conditions during data collection. The participants were supervised by the experimenters and an MRI technician during the experiment.
Timing	Data collection for one subject took place in June of 2018. Data collection for the remaining subjects took place from March of 2019 through August of 2019.
Data exclusions	Two participants were excluded from the analyses for exaggerated head motion during the scan. The motion threshold was predefined as any motion exceeding that of the voxel size (motion in any direction greater than 2.5mm). One participant was excluded due to technical difficulties during acquisition that resulted in an unusable functional image.
Non-participation	One participant dropped out of the study due to an inability to learn the stimuli required to complete the training exercise on the first day of the experiment. A second participant dropped out of the study due to an inability to remove a metal earring on the day of the MRI scan.
Randomization	Participants were not allocated into different experimental groups.

Reporting for specific materials, systems and methods

We require information from authors about some types of materials, experimental systems and methods used in many studies. Here, indicate whether each material, system or method listed is relevant to your study. If you are not sure if a list item applies to your research, read the appropriate section before selecting a response.

Materials & experimental systems

n/a	Involved in the study
☒	☐ Antibodies
☒	☐ Eukaryotic cell lines
☒	☐ Palaeontology and archaeology
☒	☐ Animals and other organisms
☒	☐ Clinical data
☒	☐ Dual use research of concern
☒	☐ Plants

Methods

n/a	Involved in the study
☒	☐ ChIP-seq
☒	☐ Flow cytometry
☐	☒ MRI-based neuroimaging

Plants

Seed stocks	NA
Novel plant genotypes	NA
Authentication	NA

Magnetic resonance imaging

Experimental design

Design type	The study employed an event-related design.
Design specifications	The task was self paced and participants completed the experiment in a single run. The main task contained 120 trials. A secondary task contained 100 trials, however, data for this trial type was not analyzed for this manuscript. Responses were followed by a 0.8s or 2.5s (randomly assigned) fixation cross before the onset of the next trial. Participants had the opportunity to pause in between game years before advancing.
Behavioral performance measures	Participants' reaction times on each trial and response accuracies were recorded. Trials were excluded from the behavioral analyses and included in a separate regressor in the GLM analyses if incorrect or if the trial reaction time was +/- 3 SD's of a participant's mean. Participants obtained a high overall accuracy (94.5% of trials remained).

Acquisition

Imaging type(s)	Structural and functional.
Field strength	7.0T
Sequence & imaging parameters	Functional data was acquired in a single run using a multi-echo multiband echo-planar imaging (EPI) pulse sequence [2.5mm isotropic resolution, 50 slices, TR=1850ms, TEs= [8.5, 23.17, 37.84, 52.51 ms], MB=2, iPAT acceleration factor=3, flip=70, field of view=640x640, pixel bandwidth=1786].
Area of acquisition	Whole brain data was acquired.

Diffusion MRI ☐ Used ☒ Not used

Preprocessing

Preprocessing software	Data preprocessing was completed using the multi-echo independent components analysis pipeline (ME-ICA). A detailed description of this pipeline can be found in Kundu et al., 2012, 2017. Additional preprocessing steps and GLM analyses were performed using FSL version 5.0.10.
Normalization	Functional images were registered to the subject-specific high resolution T1- weighted structural images using boundary based registration and to a standard brain image using a 12 DOF linear registration in FSL's fMRI Expert Analysis Tool, FEAT.
Normalization template	Images were normalized to a MNI 152 T1 template with a 2.5mm isotropic resolution.
Noise and artifact removal	The ME-ICA pipeline denoised the data for physiological and motion artifacts. Six motion parameter regressors were included in the general linear modeling analyses.
Volume censoring	No volume censoring was performed.

Statistical modeling & inference

Model type and settings	We analyzed the data using a series of general linear models implemented in FSL's FEAT. For the first-level, the primary model analysis included four goals regressors of interest, including only correct respoonses. The secondary model analysis included seven goal regressors of interest, including only correct respoonses. Primary and secondary models analyses included four regressor tracking year (objective time) in the game, and six motion parameters. First level activation maps were brought to a second level mixed effects analysis, implemented in FLAME 1 (FSL's Local Analysis of Mixed Effects), where one sample t-tests were used to determine the group mean for each contrast.
Effect(s) tested	We tested differences in blood oxygen-level-dependent (BOLD) response amplitudes elicited by the different sets of goals throughout the task. Primary analysis: two contrasts compared the temporally removed condition to the current condition (remote > current, and current > remote). Secondary analysis: eight contrasts compared activation for goals in the distant future > current, near future > current, near past > current, distant past > current, as well as the current > distant future, current > near future, current > near past, current > distant past.

Specify type of analysis: ☐ Whole brain ☐ ROI-based ☒ Both

Anatomical location(s)	The anterior and posterior parts of the hippocampus has been delimited based on Poppenk et al.'s delimitation (2013). The foci of the anterior hippocampus is defined toy = -21, using the uncal apex as the anatomical landmark.
Statistic type for inference (See Eklund et al. 2016)	All analyses were corrected for multiple comparisons implementing Family-Wise Error (FWE) using GRF-theory based maximum tresholding (voxel-wise correction, two-tailed p = 0.025).
Correction	Results were family wise error (FWE) corrected, two-tailed p = 0.025.

Models & analysis

n/a	Involved in the study
☐	☒ Functional and/or effective connectivity
☒	☐ Graph analysis
☒	☐ Multivariate modeling or predictive analysis

Functional and/or effective connectivity	*Report the measures of dependence used and the model details (e.g. Pearson correlation, partial correlation, mutual information).*
